# Optimal Resource Allocation for NOMA-TDMA Scheme with *α*-Fairness in Industrial Internet of Things

**DOI:** 10.3390/s18051572

**Published:** 2018-05-15

**Authors:** Yanjing Sun, Yiyu Guo, Song Li, Dapeng Wu, Bin Wang

**Affiliations:** 1 School of Information and Control Engineering, China University of Mining and Technology, Xuzhou 221116, China; TS16060240P3@cumt.edu.cn (Y.G.); lisong@cumt.edu.cn (S.L.); 2 School of Communication and Information Engineering, Chongqing University of Posts and Telecommunications, Chongqing 400065, China; wudp@cqupt.edu.cn; 3 Department of IoT Engineering, Xi’an University of Technology, Xi’an 710048, China; wangbin@mail.xidian.edu.cn

**Keywords:** non-orthogonal multiple access, industrial internet of things, α-fair, NOMA-TDMA scheme, NOMA-TDMA-DC algorithm

## Abstract

In this paper, a joint non-orthogonal multiple access and time division multiple access (NOMA-TDMA) scheme is proposed in Industrial Internet of Things (IIoT), which allowed multiple sensors to transmit in the same time-frequency resource block using NOMA. The user scheduling, time slot allocation, and power control are jointly optimized in order to maximize the system α -fair utility under transmit power constraint and minimum rate constraint. The optimization problem is nonconvex because of the fractional objective function and the nonconvex constraints. To deal with the original problem, we firstly convert the objective function in the optimization problem into a difference of two convex functions (D.C.) form, and then propose a NOMA-TDMA-DC algorithm to exploit the global optimum. Numerical results show that the NOMA-TDMA scheme significantly outperforms the traditional orthogonal multiple access scheme in terms of both spectral efficiency and user fairness.

## 1. Introduction

Industrial Internet of Things (IIoT) offers interconnection and interoperability to industrial systems through sensing nodes and actuators with ubiquitous communication and computation abilities [1], and has been applied in numerous areas such as industrial production, traffic monitoring, and so on [2,3,4]. As a significant component of the future transformation of industrial systems, IIoT has attracted enormous attention from academics and industries.

In conventional IIoT, sensors periodically transmit the collected data to the sink through time division multiple access (TDMA) [5]. However, TDMA only supports access of a single node in each time slot, which cause low spectrum efficiency and high transmission delay, thus it cannot meet the requirement of the IIoT with massive nodes to access [6].

Recently, the non-orthogonal multiple access (NOMA) technique has attracted significant research interest for its potential to enhance spectrum efficiency and to support different QoS requirements by allowing multiple users to transmit in the same time-frequency resource block, which is different from conventional orthogonal multiple access (OMA) techniques (e.g., TDMA, frequency division multiple access (FDMA)) [7,8,9]. To avoid the inter-user interference in NOMA network, successive interference cancellation (SIC) technique is applied to decode the received signals [10,11,12]. Inspired by the potential benefits of NOMA, many researchers have explored NOMA schemes in different kinds of communications, such as cellular IoT, machine-to-machine communications and cooperation communications [13,14,15]. Several works have been carried out on performance analysis and optimization of NOMA, including spectral efficiency, energy efficiency, physical layer security, and visible light communications [16,17,18,19]. In [16], the resource allocation for multicarrier NOMA system with full-duplex base station and multiple half-duplex downlink and uplink users is explored. where both optimal and suboptimal schemes are proposed to maximize the weighted throughput. In [17], the machine-to-machine communication for uplink NOMA system is studied to minimize the total energy consumption of the network. In [18], the physical layer security issue is investigated in NOMA system. The secrecy sum rate of NOMA system is maximized and the closed-form of optimal power is derived.

As a promising multiple access scheme proposed in 5G, NOMA is superior to the conventional multiple access scheme in the massive uplink scenario of IIoT where massive nodes transmit small data packets. The technical features of NOMA enable it to support more users or devices than conventional orthogonal multiple access technologies (TDMA, FDMA, OFDMA) and are more suited for IIoT, which has a large number of connections and low speed requirements [20]. Therefore, we investigate NOMA mechanism in IIoT in this paper, considering fairness of system.

The commonly used fairness models include proportional fairness, max-min fairness, and α -fairness. However, the proportional fairness and max-min fairness can only achieve absolute fairness, which means that all users have the same performance (e.g., the same outage probability or the same ergodic rate) [21,22,23]. α -fairness only utilizes a single variable α to achieve different well-known efficiency-fairness tradeoffs and user fairness levels [24], including sum-rate maximization, proportional fairness, and max-min fairness. It is suitable for IIoT with massive sensors, because as a unified fairness model, α -fairness can achieve different fairness levels for different sensors without employing different fairness models. The relationship between efficiency and fairness in the system is expressed by α -fair utility.

Considering the performance improvement achieved by NOMA, in this paper we propose a joint non-orthogonal multiple access and time division multiple access (NOMA-TDMA) scheme in IIoT considering α -fairness. In our system, the transmission time is divided into multiple time slots with different length in TDMA scheme and each time slot can be assigned to one or more nodes who complete their transmissions in NOMA scheme. The main contributions of this paper are summarized as follows:

(1) A NOMA-TDMA communication scheme considering α -fairness is proposed in IIoT, which allows multiple sensors to transmit data through NOMA in each time slot and total available transmission time is allocated for all nodes in TDMA scheme.
(2) Incorporating user scheduling, power allocation and time slot assignment based on the NOMA-TDMA scheme, an optimization problem is formulated to maximize the α -fair utility of the IIoT system under the constraint on sensors’ transmit rate and constraint on aggregate power. To tackle the resource allocation problem, the original optimal problem is transformed into a D.C. structure by variable substitution. Furthermore, we proposed an algorithm regarded as NOMA-TDMA-DC, which can quickly find global optimal solution.

## 2. System Model and Problem Formulation

### 2.1. System Model

**Figure 1:**
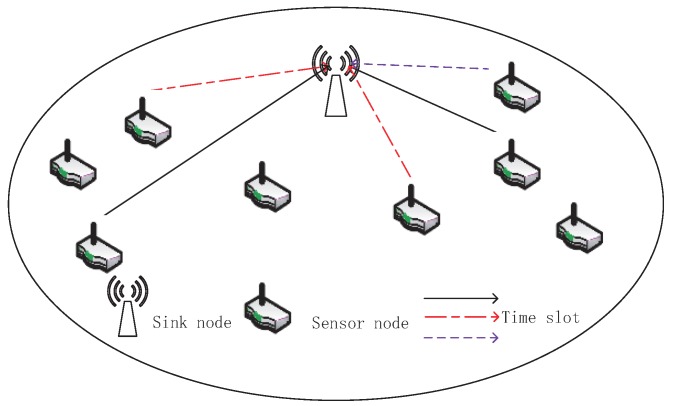
System model of uplink IIoT.

Consider an uplink IIoT scenario with one sink and N sensors as shown in Figure 1. The channel fading coefficient from the sensor *i* to the sink is denoted by $h_{i}$ . Without loss of generality, it is assumed that $\left|h_{1}\right|\geq \left|h_{2}\right|\geq \ldots \geq \left|h_{N}\right|$ . Multiple sensors can transmit data to the sink using NOMA to enhance spectrum efficiency and acceptable concurrent nodes number. Considering the computing ability of the sink in IIoT, the decoding complexity, and the possible delay of arrival signal, we only focus on the case that two sensors are associated with NOMA. In the following, between the two sensors that are paired in NOMA, the sensor with a poor channel fading coefficient is regarded as “weak sensor”, and the other one is regarded as “strong sensor”.

### 2.2. NOMA-TDMA Scheme

**Figure 2:**
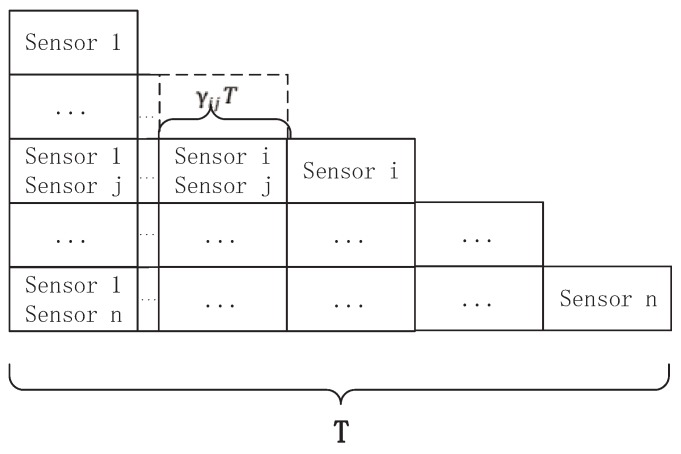
The time slot assignment of NOMA-TDMA scheme.

The proposed NOMA-TDMA scheme is described as follows: The available transmission time *T* is divided into N∗(N/2+1) time slots, each of which is identified by a two-dimensional vector (i,j) and denoted as $T_{ij}$ . In time slot $T_{ij}$ , sensor *i* and sensor *j* send data to the sink using NOMA. When i=j , sensor *i* exclusively occupies this slot and sends data to the sink in time slot $T_{ii}$ . The length of the time slot $T_{ij}$ is $\gamma_{ij}T$ , where $\sum_{i=1}^{N}\sum_{j=i}^{N}\gamma_{ij}\leq 1\left\{\forall i\in N,\forall j\in N|\gamma_{ij}\geq 0\right\}$ . As shown in Figure 2.

### 2.3. Problem Formulation

According to the NOMA-TDMA scheme, sensor *i* and sensor *j* transmit their messages $\left(i<j\right)$ over the same channel using NOMA with transmission powers $p_{i}$ and $p_{j}$ , respectively. The received signal *y* at sink can be represented as

(1) $$y=\sqrt{p_{i}}h_{i}x_{i}+\sqrt{p_{j}}h_{j}x_{j}+n_{0}$$

where $x_{i}$ and $x_{j}$ are the messages transmitted by sensor *i* and sensor *j*, respectively. The NOMA scheme is designed such that the messages can be reliably extracted from the combined message y. The sink decode $x_{j}$ firstly and the received Signal to Interference plus Noise Ratio (SINR) can be expressed as


(2) $$SINR_{j}=\frac{{\left|h_{j}\right|}^{2}p_{j}}{{\left|h_{i}\right|}^{2}p_{j}+n_{0}}.$$

Then the sink employs successive interference cancellation (SIC) to deduct the successfully decoded message $x_{j}$ from the received *y* before decoding $x_{i}$ . Hence, the received SINR of sensor *i* is given by


(3) $$SINR_{i}=\frac{{\left|h_{i}\right|}^{2}p_{i}}{N_{0}}.$$

The achievable data rate of the sensor *i* in the available transmission time *T* can be expressed according to the sum rate of the sensor *i* pairing with other nodes


(4) $$R^{\left(i\right)}=T*\left(\sum_{k=1}^{i-1}\gamma_{ki}R_{ki}^{\left(i\right)}+\sum_{j=i+1}^{N}\gamma_{ij}R_{ij}^{\left(i\right)}+\gamma_{ii}R_{ii}\right).$$

In the first term, $R_{ki}^{\left(i\right)}$ denotes the rate of sensor *i* as the weak sensor in time slot $T_{ki}\left(k<i\right)$ , in which sensor *i* and sensor *k* transmit their signals in NOMA scheme. In the second term, $R_{ij}^{\left(i\right)}$ denotes the rate of sensor *i* as the strong sensor in time slot $T_{ij}\left(i<j\right)$ , in which sensor *i* and sensor *j* transmit their signals in NOMA scheme. In the third term, $R_{ii}$ denotes the rate of sensor *i* transmit alone in $T_{ii}$ time slot. The rate of each situation can be expressed respectively as

(5) $$R_{ki}^{\left(i\right)}=\log_{2}\left(1+\frac{p_{ki}^{\left(i\right)}{\left|h_{i}\right|}^{2}}{n_{0}+p_{ki}^{\left(k\right)}{\left|h_{i}\right|}^{2}}\right)$$

(6) $$R_{ij}^{\left(i\right)}=\log_{2}\left(1+\frac{p_{ij}^{\left(i\right)}{\left|h_{i}\right|}^{2}}{n_{0}}\right)$$

(7) $$R_{ii}=\log_{2}\left(1+\frac{p_{ii}{\left|h_{i}\right|}^{2}}{n_{0}}\right)$$

where $p_{ki}^{\left(i\right)}$ , $p_{ij}^{\left(i\right)}$ and $p_{ii}$ denote the transmission power of sensor *i* in time slot $T_{ki}$ , $T_{ij}$ and $T_{ii}$ , respectively.

According to each sensor’s QoS requirement, transmit rate should not be less than a certain threshold $R^{req}$ , i.e.,


(8) $$R_{i}\geq R^{req}\;\forall i\in N.$$

In addition, the transmission power of each sensor cannot exceed a certain maximum transmission power $P_{max}$ . Hence, we have


(9) $$0\leq p_{ij}^{i}\leq p_{max}\;\forall i,j\in N.$$

We denote the α -fair utility function as $U_{\alpha }\left(x\right)\left(\alpha \geq 0\right)$ , which can be expressed as [25]


(10) $$U_{\alpha }=\left\{\begin{array}{cc} \ln(x) & if\;\alpha =1\\ \frac{1}{1-\alpha }x^{1-\alpha } & otherwise \end{array}\right.$$

Assume that sensor *i* receives utility $U_{\alpha }\left(R^{\left(i\right)}\right)$ when its received rate is $R^{\left(i\right)}$ . We should note that the optimization problem with α=0 , α=1 , and α=∞ just correspond to sum-rate maximization, proportional fairness, and max-min fairness, respectively [26], and we only focus on the condition that 0<α<1 in this paper for it is fair enough in IIoT when the α = 1.

We aim at maximizing the sum α -fair utility of system while guaranteeing each user’s QoS by joint user scheduling, time slot allocation, and power control. Then, the optimization problem can be formulated as

(11a) $$\;\begin{array}{cc} \max_{\gamma ,p} & \;\sum_{i=1}^{N}{\frac{1}{1-\alpha }\left(T*\left(\sum_{k=1}^{i-1}\gamma_{ki}R_{ki}^{\left(i\right)}+\sum_{j=i+1}^{N}\gamma_{ij}R_{ij}^{\left(i\right)}+\gamma_{ii}R_{ii}\right)\right)}^{1-\alpha } \end{array}$$

(11b) $$\begin{array}{cc} \;\mathrm{subject}\;\mathrm{to} & \;C1\text{:}0\leq p_{ij}^{i}\leq p_{max}\;\forall i,j\in N \end{array}$$

(11c) $$\begin{array}{c} \;C2\text{:}R^{\left(i\right)}\geq R^{req}\;\;\;\;\forall i\in N \end{array}$$

(11d) $$\begin{array}{c} \;C3\text{:}\sum_{i=1}^{N}\sum_{j=i}^{N}\gamma_{ij}\leq 1 \end{array}$$

where (11b) and (11c) denote that the sensor’s QoS constraints and maximum transmit power constraints, respectively. (11d) denotes that available transmission time constraints allocated to all sensors.

## 3. Optimal Resource Allocation

In order to handle the situation where the sensor’s rate requirement is zero, we first present a remark about the optimal solution of the problem in (11a).

**Remark** **1.** *Even if $R^{req}=0$ for all sensors, the optimal rate of each sensor is always greater than zero for 0<α<1 .*

**Proof.**  Because the derivative of $U_{\alpha }\left(x\right)$ is infinite at x=0 and finite at x>0 , there is always a positive value C in $\sum_{i=1}^{N}U_{\alpha }\left(R^{\left(i\right)}\right)$ such that the optimal rate of sensor *i* is not less than C. ☐

According to Remark 1, in the remainder of this paper, we replace constraint C2 of problem (11b) by $C2{}^{\prime }$ given by

(12) $${C2}^{\prime }\text{:}R^{\left(i\right)}\geq C\;\;\;\;\forall i\in N,$$

where C>0 and the value of *C* is discussed as follows.

When $R^{req}>0$ , $C=R^{req}$ . In this case, constraint C2′ and C2 are equivalent, and therefore, the optimality has not changed.

When $R^{req}=0$ , *C* equals a very small positive value. According to Remark 1, the optimality also has not changed.

In the following, we first transform the objective function of the problem in (11a) into a D.C. function. Then, an algorithm is designed, followed by the discussion of the validity, optimality, convergence, parameter setting, and the expansion of the NOMA-TDMA-DC.

### 3.1. Transformation of the Objective Function

The rate expression of $R^{\left(i\right)}$ , which is non-convex, is first converted to the D.C. form. Denote ${\hat{p}}_{ki}^{\left(i\right)}=\gamma_{ki}p_{ki}^{\left(i\right)}$ , ${\hat{p}}_{ij}^{\left(i\right)}=\gamma_{ij}p_{ij}^{\left(i\right)}$ and ${\hat{p}}_{ii}^{\left(i\right)}=\gamma_{ii}p_{ii}^{\left(i\right)}$ , then we can get a new variable set $\hat{p}$ and $\gamma_{ki}p_{ki}^{\left(i\right)}$ , $\gamma_{ij}p_{ij}^{\left(i\right)}$ and $\gamma_{ii}p_{ii}^{\left(i\right)}$ can be converted to

(13) $$\begin{array}{cc} L_{1,ki}-K_{1,ki} & =\gamma_{ki}R_{ki}^{\left(i\right)}\\ & =\gamma_{ki}\log_{2}\left(1+\frac{\frac{{\hat{p}}_{ki}^{\left(k\right)}{\left|h_{i}\right|}^{2}}{n_{0}}}{\gamma_{ki}}+\frac{\frac{{\hat{p}}_{ki}^{\left(i\right)}{\left|h_{i}\right|}^{2}}{n_{0}}}{\gamma_{ki}}\right)-\gamma_{ki}\log_{2}\left(1+\frac{\frac{{\hat{p}}_{ki}^{\left(k\right)}{\left|h_{i}\right|}^{2}}{n_{0}}}{\gamma_{ki}}\right), \end{array}$$

(14) $$L_{2,ki}=\gamma_{ij}R_{ij}^{\left(i\right)}=\gamma_{ij}\log_{2}\left(1+\frac{\frac{{\hat{p}}_{ij}^{\left(i\right)}{\left|h_{i}\right|}^{2}}{n_{0}}}{\gamma_{ij}}\right),$$

(15) $$L_{3,ki}=\gamma_{ii}R_{ii}^{\left(i\right)}=\gamma_{ii}\log_{2}\left(1+\frac{\frac{{\hat{p}}_{ii}^{\left(i\right)}{\left|h_{i}\right|}^{2}}{n_{0}}}{\gamma_{ii}}\right).$$

We can easily see that $L_{1,ki}$ , $L_{2,ij}$ , $L_{3,ii}$ and $k_{1,ki}$ are concave on $\hat{p}$ and γ . Then the rate of sensor *i* can be expressed as

(16) $$R^{\left(i\right)}=u_{i}\left(\hat{p},\gamma \right)-v_{i}\left(\hat{p},\gamma \right),$$

where both $u_{i}\left(\hat{p},\gamma \right)$ and $v_{i}\left(\hat{p},\gamma \right)$ are concave given by


(17) $$u_{i}\left(\hat{p},\gamma \right)=L_{1,ki}+L_{2,ij}+L_{3,ii},$$

(18) $$v_{i}\left(\hat{p},\gamma \right)=K_{1,ki}.$$

The constraints $C2{}^{\prime }$ has been transformed to a D.C. function, and now we focus on the objective function (11a) which can be expressed as

(19) $$U_{\alpha }\left(R^{\left(i\right)}\right)=U_{\alpha }\left(u_{i}\left(\hat{p},\gamma \right)-v_{i}\left(\hat{p},\gamma \right)\right)=g_{i}\left(\hat{p},\gamma \right)-h_{i}\left(\hat{p},\gamma \right),$$

where $g_{i}\left(\hat{p},\gamma \right)$ and $h_{i}\left(\hat{p},\gamma \right)$ are given by

(20) $$g_{i}\left(\hat{p},\gamma \right)=U_{\alpha }\left(R^{\left(i\right)}\right)+Z_{i}v_{i}\left(\hat{p},\gamma \right),$$

(21) $$h_{i}\left(\hat{p},\gamma \right)=Z_{i}v_{i}\left(\hat{p},\gamma \right)$$

respectively. It can be easily deduced that $U_{\alpha }\left(R^{\left(i\right)}\right)=g_{i}\left(\hat{p},\gamma \right)-h_{i}\left(\hat{p},\gamma \right)$ and $Z_{i}$ is a constant that is greater than or equal to $\frac{1}{C^{\alpha }}$ . According to Remark 1, the value of *C* is always greater than zero.

To prove that (19) is a D.C. function, we need to prove that both $g_{i}\left(\hat{p},\gamma \right)$ and $h_{i}\left(\hat{p},\gamma \right)$ are concave. Since $v_{i}\left(\hat{p},\gamma \right)$ is a concave function and $Z_{i}$ is a positive scalar, $h_{i}\left(\hat{p},\gamma \right)$ is a concave function. Then, in order to prove that $g_{i}\left(\hat{p},\gamma \right)$ is also a concave function, we present Theorem 1.

**Theorem** **1.** *
$g_{i}\left(\hat{p},\gamma \right)$ given by (20) is concave.*

**Proof.**  First, note that $U_{\alpha }\left(t\right)$ is a concave function at $\left[C,+\infty \right)$ and its derivative is $\frac{1}{t^{\alpha }}$ , so we have

(22) $$U_{\alpha }\left(t\right)\leq U_{\alpha }\left(\theta \right)+\frac{1}{\theta^{\alpha }}\left(t-\theta \right)\;t\geq C$$

for all $\theta \in \left[C,+\infty \right)$ and is equal at t=θ . Therefore, $U_{\alpha }\left(t\right)$ can be written as

(23) $$U_{\alpha }\left(t\right)=\underset{\theta \in \left[C,+\infty \right)}{\inf}\left\{U_{\alpha }\left(\theta \right)+\frac{1}{\theta^{\alpha }}\left(t-\theta \right)\right\}=\underset{\theta \in \left[C,+\infty \right)}{\inf}\left\{U_{\alpha }\left(\theta \right)+\frac{t}{\theta^{\alpha }}-\theta^{1-\alpha }\right\}.$$

Substituting t for $U_{\alpha }\left(u_{i}\left(\hat{p},\gamma \right)-v_{i}\left(\hat{p},\gamma \right)\right)$ , we can get

(24) $$U_{\alpha }\left(u_{i}\left(\hat{p},\gamma \right)-v_{i}\left(\hat{p},\gamma \right)\right)=\underset{\theta \in \left[C,+\infty \right)}{\inf}\left\{U_{\alpha }\left(\theta \right)+\frac{u_{i}\left(\hat{p},\gamma \right)-v_{i}\left(\hat{p},\gamma \right)}{\theta^{\alpha }}-\theta^{1-\alpha }\right\}.$$

Hence, (20) can be formulated as:

(25) $$\begin{array}{cc} g_{i}\left(\hat{p},\gamma \right) & =U_{\alpha }\left(R^{\left(i\right)}\left(\hat{p},\gamma \right)\right)+Z_{i}v_{i}\left(\hat{p},\gamma \right)\\ & =\underset{\theta \in \left[C,+\infty \right)}{\inf}\left\{U_{\alpha }\left(\theta \right)+\frac{u_{i}\left(\hat{p},\gamma \right)-v_{i}\left(\hat{p},\gamma \right)}{\theta^{\alpha }}-\theta^{1-\alpha }\right\}+Z_{i}v_{i}\left(\hat{p},\gamma \right)\\ & =\underset{\theta \in \left[C,+\infty \right)}{\inf}\left\{U_{\alpha }\left(\theta \right)+\frac{u_{i}\left(\hat{p},\gamma \right)}{\theta^{\alpha }}+\left(Z_{i}-\frac{1}{\theta^{\alpha }}\right)v_{i}\left(\hat{p},\gamma \right)-\theta^{1-\alpha }\right\}. \end{array}$$

Since $Z_{i}\geq \frac{1}{C^{\alpha }}\geq \frac{1}{\theta^{\alpha }}$ for all $\theta \in \left[C,+\infty \right)$ , and both $u_{i}\left(\hat{p},\gamma \right)$ and $v_{i}\left(\hat{p},\gamma \right)$ are concave, $g_{i}\left(\hat{p},\gamma \right)$ can be regarded as the infimum of an infinite set of concave functions and is, thus, concave.

Therefore, we complete the proof. ☐

According to Theorem 1, it can be concluded that $U_{\alpha }\left(R^{\left(i\right)}\right)=g_{i}\left(\hat{p},\gamma \right)-h_{i}\left(\hat{p},\gamma \right)$ is a D.C. function, and $U_{\alpha }\left(R^{\left(i\right)}\right)$ can be written as

(26) $$\begin{array}{cc} \sum_{i=1}^{N}U_{\alpha }\left(R^{\left(i\right)}\right) & =\sum_{i=1}^{N}\left(g_{i}\left(\hat{p},\gamma \right)-h_{i}\left(\hat{p},\gamma \right)\right)\\ & =\sum_{i=1}^{N}g_{i}\left(\hat{p},\gamma \right)-\sum_{i=1}^{N}h_{i}\left(\hat{p},\gamma \right)\\ & =g\left(\hat{p},\gamma \right)-h\left(\hat{p},\gamma \right) \end{array}$$

where $\sum_{i=1}^{N}g_{i}\left(\hat{p},\gamma \right)$ and $\sum_{i=1}^{N}h_{i}\left(\hat{p},\gamma \right)$ are concave. The objective function (11a) and the constraint (12) can be expressed by the D.C. structure.

### 3.2. NOMA-TDMA Based on D.C. Programming (NOMA-TDMA-DC)

In this section, NOMA-TDMA-DC algorithm is proposed to solve the optimization problem. First, we perform the first-order Taylor expansion of h(p,γ) , i.e., $h\left({\hat{p}}^{\varepsilon },\gamma^{\varepsilon }\right)+\nabla h^{T}\left({\hat{p}}^{\varepsilon },\gamma^{\varepsilon }\right){\left(\left[\hat{p},\gamma \right]-\left[{\hat{p}}^{\varepsilon },\gamma^{\varepsilon }\right]\right)}^{T}$ . Similarly, the first-order Taylor expansion of $v_{i}\left(p,\gamma \right)$ is performed. Then the initial point $\left[{\hat{p}}^{\varepsilon },\gamma^{\varepsilon }\right]$ for the ε iteration is set and $\left[{\hat{p}}^{\varepsilon +1},\gamma^{\varepsilon +1}\right]$ is optimized in the ε+1 iteration, which is obtained by solving the problem P1.


(27a) $$\begin{array}{cc} P1\text{:} & \;\max_{\gamma ,p}\;g\left(\hat{p},\gamma \right)-\left(h\left({\hat{p}}^{\varepsilon },\gamma^{\varepsilon }\right)+\nabla h^{T}\left({\hat{p}}^{\varepsilon },\gamma^{\varepsilon }\right){\left(\left[\hat{p},\gamma \right]-\left[{\hat{p}}^{\varepsilon },\gamma^{\varepsilon }\right]\right)}^{T}\right) \end{array}$$

(27b) $$\;\begin{array}{cc} s.t. & \;{\hat{p}}_{i,j}^{\left(i\right)}-\gamma_{i,j}P_{max}\leq 0\;\forall i,j\in N \end{array}$$

(27c) $$\;\begin{array}{c} u_{i}\left(\hat{p},\gamma \right)-\left(v_{i}\left({\hat{p}}^{\varepsilon },\gamma^{\varepsilon }\right)+\nabla v_{i}^{T}\left({\hat{p}}^{\varepsilon },\gamma^{\varepsilon }\right){\left(\left[\hat{p},\gamma \right]-\left[{\hat{p}}^{\varepsilon },\gamma^{\varepsilon }\right]\right)}^{T}\right)\geq C\;\forall i\in N \end{array}$$

(27d) $$\;\begin{array}{c} \sum_{i=1}^{N}\sum_{j=i}^{N}\gamma_{ij}\leq 1 \end{array}$$

(27e) $$\begin{array}{c} \hat{p}\geq 0\;\gamma \geq 0 \end{array}$$

After Taylor expansion on the initial point, (27a) and (27c) are convex, so P1 is convex optimization problem and can be solved by interior point method.

The detailed process of NOMA-TDMA-DC is summarized in Algorithm 1, where ε is the iterative index of the algorithm , $B^{\varepsilon }$ is the sum α -fair utility of system in the ε th iterative and δ is the convergence accuracy of the algorithm.

**Algorithm 1** NOMA-TDMA-DC**Initialization:**
  Set the initial point 
$p^{0}$
, 
$\gamma^{0}$
, and 
δ
;  
ε
 = 0; 1: 
**repeat** 2: Solve the convex optimization problem P1 to obtain the solution 
$\left[p^{*},\gamma^{*}\right]$
;   
ε=ε+1
;   
$\left[p^{\varepsilon },\gamma^{\varepsilon }\right]=\left[p^{*},\gamma^{*}\right]$
; 3: **until**

$\left|B^{\varepsilon }-B^{\varepsilon +1}\right|\leq \delta$


### 3.3. Algorithm Performance Analysis

The validity, convergence and complexity of the NOMA-TDMA-DC discussed in this section.

Validity: In each iteration of the NOMA-TDMA-DC, the second concave function approximating the objective function, i.e., $h(\hat{p},\gamma )$ , is expanded by its first-order Taylor expansion. Because of its logarithmic structure, $h(\hat{p},\gamma )$ is well approximated by its first-order Taylor expansion in the relatively large neighborhood of $\left[{\hat{p}}^{\varepsilon },\gamma^{\varepsilon }\right]$ due to its low sensitivity to the variation of the variable $\left(\hat{p},\gamma \right)$ . Therefore, the non-convex optimization problem (11a) is well approximated by the convex optimization problem (27a). In mathematical essence, the transformation of optimal problem of in [27,28] is the same as (27a).

Convergence: The NOMA-TDMA-DC always converges to the optimal point of the original resource allocation problem (11a), whose problem of each iteration is convex and can be solved by the interior point method. In the NOMA-TDMA-DC, the last step follows from the concavity of $h(\hat{p},\gamma )$ . Thus, for any given $\left({\hat{p}}^{\varepsilon },\gamma^{\varepsilon }\right)$ , we have

(28) $$\begin{array}{c} g\left({\hat{p}}^{\varepsilon },\gamma^{\varepsilon }\right)-h\left({\hat{p}}^{\varepsilon },\gamma^{\varepsilon }\right)\\ \leq g\left({\hat{p}}^{\varepsilon +1},\gamma^{\varepsilon +1}\right)-\left(h\left({\hat{p}}^{\varepsilon },\gamma^{\varepsilon }\right)+\nabla h^{T}\left({\hat{p}}^{\varepsilon },\gamma^{\varepsilon }\right){\left(\left[{\hat{p}}^{\varepsilon +1},\gamma^{\varepsilon +1}\right]-\left[{\hat{p}}^{\varepsilon },\gamma^{\varepsilon }\right]\right)}^{T}\right)\\ \leq g\left({\hat{p}}^{\varepsilon +1},\gamma^{\varepsilon +1}\right)-h\left({\hat{p}}^{\varepsilon +1},\gamma^{\varepsilon +1}\right). \end{array}$$

Hence, the solution of (27a) is always improved after each iteration and due to the compactness of the constraint set, the improved solution sequence $\left({\hat{p}}^{\varepsilon },\gamma^{\varepsilon }\right)$ always converges.

Complexity: In our system, the sensors transmit their channel fading coefficients to the sink and the main computation process is implemented in a centralized manner at the sink. After deriving the optimization results, the sink broadcasts the necessary control information to sensors. The computational complexity of NOMA-TDMA-DC includes two parts: the computational complexity of each iteration and the number of iterations required for algorithm convergence. In each iteration, the interior point method is employed with the computational complexity of $O(M^{3})$ , where M is the number of variables in (11a) as $M=N^{2}+2N$ . On the other hand, since $g(\hat{p},\gamma )$ and $h(\hat{p},\gamma )$ are all concave piecewise linear, the NOMA-TDMA-DC converges linearly with a complexity $O(\log\frac{1}{\delta })$ , where δ is the convergence accuracy of the algorithm.Therefore, the NOMA-TDMA-DC has the complexity of $O(M^{3}\log\frac{1}{\delta })$ .

### 3.4. Discussion on the Value of $Z_{i}$

In this subsection, we analyze the impact of $Z_{i}$ on the solution to problem (27a).

$Z_{i}$ needs to be large enough to guarantee the convexity of $g_{i}\left(\hat{p},\gamma \right)$ given in (25), meanwhile $Z_{i}$ cannot be too large for the following reason. In the ε+1 iteration, the objective function is

(29) $$\begin{array}{c} g\left({\hat{p}}^{\varepsilon +1},\gamma^{\varepsilon +1}\right)\;-\;\left(h\left({\hat{p}}^{\varepsilon },\gamma^{\varepsilon }\right)\;+\;\nabla h^{T}\left({\hat{p}}^{\varepsilon },\gamma^{\varepsilon }\right){\left(\left[{\hat{p}}^{\varepsilon +1},\gamma^{\varepsilon +1}\right]\;-\;\left[{\hat{p}}^{\varepsilon },\gamma^{\varepsilon }\right]\right)}^{T}\right)\\ =\sum_{i=1}^{N}U_{\alpha }\left(R^{\left(i\right)}\right)\;+\;Z_{i}\left(v_{i}\left({\hat{p}}^{\varepsilon +1},\gamma^{\varepsilon +1}\right)\;-\;v_{i}\left({\hat{p}}^{\varepsilon },\gamma^{\varepsilon }\right)\;-\;\nabla v_{i}^{T}\left({\hat{p}}^{\varepsilon },\gamma^{\varepsilon }\right){\left(\left[{\hat{p}}^{\varepsilon +1},\gamma^{\varepsilon +1}\right]\;-\;\left[{\hat{p}}^{\varepsilon },\gamma^{\varepsilon }\right]\right)}^{T}\right). \end{array}$$

$Z_{i}\left(v_{i}\left({\hat{p}}^{\varepsilon +1},\gamma^{\varepsilon +1}\right)-v_{i}\left({\hat{p}}^{\varepsilon },\gamma^{\varepsilon }\right)-\nabla v_{i}^{T}\left({\hat{p}}^{\varepsilon },\gamma^{\varepsilon }\right){\left(\left[{\hat{p}}^{\varepsilon +1},\gamma^{\varepsilon +1}\right]-\left[{\hat{p}}^{\varepsilon },\gamma^{\varepsilon }\right]\right)}^{T}\right)$ will be the main part of the objective function when $Z_{i}$ is too large. Because $v_{i}\left(\hat{p},\gamma \right)$ is concave on $\left[\hat{p},\gamma \right]$ , there is $v_{i}\left({\hat{p}}^{\varepsilon +1},\gamma^{\varepsilon +1}\right)-v_{i}\left({\hat{p}}^{\varepsilon },\gamma^{\varepsilon }\right)-\nabla v_{i}^{T}\left({\hat{p}}^{\varepsilon },\gamma^{\varepsilon }\right){\left(\left[{\hat{p}}^{\varepsilon +1},\gamma^{\varepsilon +1}\right]-\left[{\hat{p}}^{\varepsilon },\gamma^{\varepsilon }\right]\right)}^{T}\leq 0$ , equivalent if and only if $\left[{\hat{p}}^{\varepsilon +1},\gamma^{\varepsilon +1}\right]=\left[{\hat{p}}^{\varepsilon },\gamma^{\varepsilon }\right]$ . Hence, optimizing (27a) when $Z_{i}$ is too large leads to $\left[{\hat{p}}^{\varepsilon +1},\gamma^{\varepsilon +1}\right]$ very close to $\left[{\hat{p}}^{\varepsilon },\gamma^{\varepsilon }\right]$ , which means algorithm convergence will be very slow. So we set $Z_{i}=\frac{1}{C^{\alpha }}$ .

## 4. Simulation Results and Discussion

In this section, the performance of the proposed NOMA-TDMA scheme is evaluated through simulations, where we set the noise as an additive white Gaussian variable with zero mean and unit variance, and the transmit signal-to-noise-ratio (SNR) is equivalent to the transmit power. Furthermore, we utilize Jain’s index to evaluate the degree of system fairness, which is defined as [29]:

(30) $$\mathrm{Jain}’s\;\mathrm{Index}=\frac{{\left(\sum_{i=1}^{N}R_{i}\right)}^{2}}{N\sum_{i=1}^{N}{R_{i}}^{2}}.$$

The result is between $\left[\frac{1}{N},1\right]$ and a larger Jain’s index indicates that the system is more fair.

**Figure 3:**
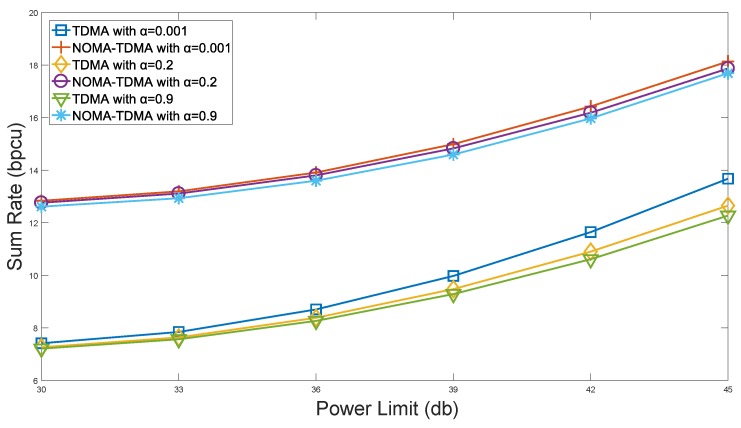
Performance of system sum rate with power constraints.

Figure 3 shows the IIoT sum rate with different power constraints, where the number of sensor in system is 20 and minimum rate requirement of each sensor is 0.5b/s . From Figure 3, the proposed NOMA-TDMA scheme is significantly better than TDMA scheme, since NOMA allows two sensors transmitting at the same time-frequency resource block, which enhances the spectrum efficiency and improve the throughput of the system. Furthermore, we can see that the curves of NOMA-TDMA scheme are very close even for different values of α . The transmission of a strong sensor is always accompanied by the transmission of a weak sensor and this transmission scheme guarantees the fairness in the system, thus NOMA-TDMA scheme is less affected by the system fairness requirements than the TDMA scheme.

**Figure 4:**
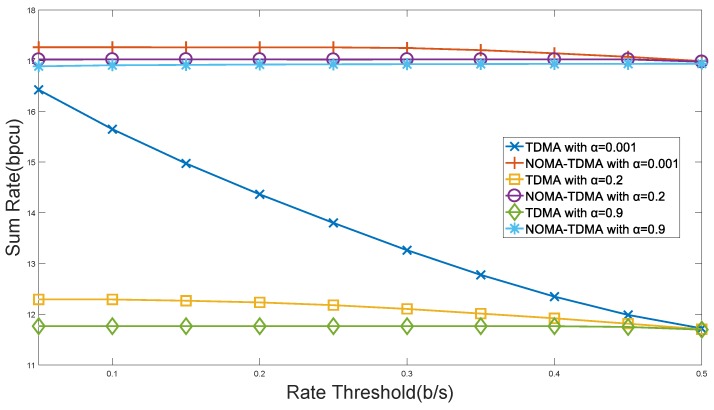
Performance of system sum rate with rate constraints.

Figure 4 depicts the IIoT sum rate with different rate thresholds of sensors. It can be seen that, compared with the TDMA scheme, the proposed NOMA-TDMA scheme is more suitable for the IIoT scenario with massive sensors access. The TDMA scheme will allocate resources to the sensors which have better transmission conditions, and the performance of the system will be significantly reduced as the rate threshold of each sensor increases. While the NOMA-TDMA scheme allows the sink to serve the sensor that needs to meet the minimum rate requirement and the sensor that has a good channel gain at the same resource block, which will increase the sum rate satisfying the rate requirement of each sensor. Therefore, the IIoT will be less affected by the rate threshold and be more stable.

**Figure 5:**
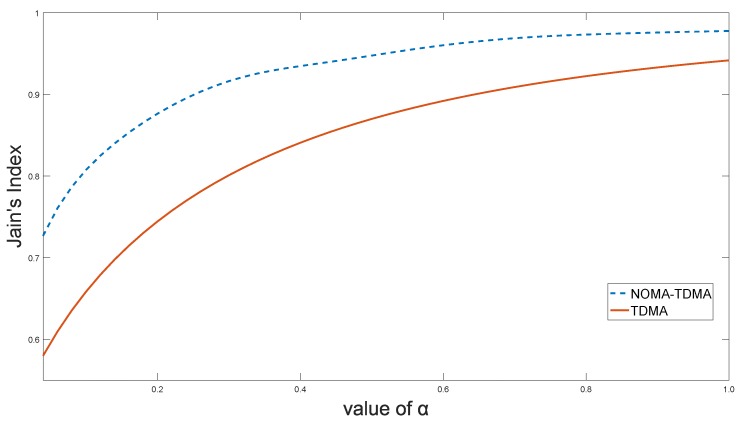
Jain’s Index versus the value of α .

Figure 5 shows the relationship between the Jain’s Index and the value of α . As can be observed, Jain’s Index and the value of α are positively related, indicating that the degree of IIoT fairness is increasing with the increase of α . The Jain’s Index can reach 0.9964 when α=1 by using the proposed NOMA-TDMA scheme, which can meet the fairness requirements of most IIoT scenarios. Furthermore, compared to other fairness models, α -fairness provides a smooth tradeoff between system sum rate and fairness.

**Figure 6:**
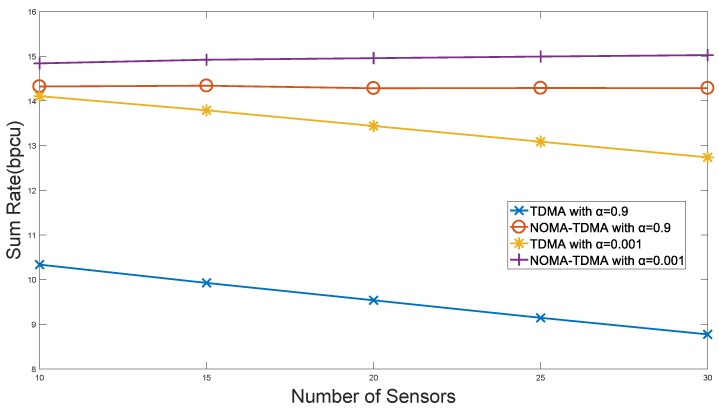
Performance of system sum rate with number of sensors.

Figure 6 presents the IIoT sum rate with different access number. We can see that as the number of sensors increases, the sum rate will drop significantly, since the TDMA scheme needs limited resources to be allocated to more sensors. Benefiting from the multi-user diversity gain of the NOMA-TDMA scheme, IIoT sum rate will increase in a certain range with the number of sensors, both in the case of α=0.9 that emphasizes fairness, and α=0.001 that emphasizes throughput. This indicates that the NOMA-TDMA is more suitable for massive sensors access IIoT scenarios.

## 5. Conclusions

In this paper, we proposed a NOMA-TDMA scheme for IIoT scenarios, which allowed multiple sensors to transmit in the same time-frequency resource block. To maximize the sum α -fair utility of system, a joint user scheduling, time slot allocation and power control problem is formulated. Furthermore, the original non-convex optimization problem is converted into an equivalent D.C. problem and NOMA-TDMA-DC algorithm is developed, which can converge to the optimal solution under a limited iterations. The simulation results showed that the proposed NOMA-TDMA scheme outperforms the OMA scheme in terms of fairness and throughput; it also improves the spectrum efficiency, indicating that the NOMA-TDMA scheme has a better application prospect in the IIoT.
